# Dissecting *in Vitro* the Activation of Human Immune Response Induced by *Shigella sonnei* GMMA

**DOI:** 10.3389/fcimb.2022.767153

**Published:** 2022-02-03

**Authors:** Serena Tondi, Bruna Clemente, Carmen Esposito, Chiara Sammicheli, Simona Tavarini, Laura B. Martin, Omar Rossi, Francesca Micoli, Erika Bartolini, Michela Brazzoli, Cristina Ulivieri, Christoph J. Blohmke, Francesca Schiavetti

**Affiliations:** ^1^ GlaxoSmithKline (GSK), Preclinical Evidence Generation (PEG), Siena, Italy; ^2^ Department of Life Sciences, University of Siena, Siena, Italy; ^3^ GlaxoSmithKline (GSK) Vaccines Institute for Global Health S.R.L. (GVGH), Siena, Italy; ^4^ GlaxoSmithKline (GSK), Artificial Intelligence & Machine Learning (AIML), London, United Kingdom

**Keywords:** GMMA, Shigella sonnei, vaccines, immune response, hPBMCs, *in vitro*, OMV

## Abstract

Generalized Modules for Membrane Antigens (GMMA) are outer membrane exosomes purified from Gram-negative bacteria genetically mutated to increase blebbing and reduce risk of reactogenicity. This is commonly achieved through modification of the lipid A portion of lipopolysaccharide. GMMA faithfully resemble the bacterial outer membrane surface, and therefore represent a powerful and flexible platform for vaccine development. Although GMMA-based vaccines have been demonstrated to induce a strong and functional antibody response in animals and humans maintaining an acceptable reactogenicity profile, the overall impact on immune cells and their mode of action are still poorly understood. To characterize the GMMA-induced immune response, we stimulated human peripheral blood mononuclear cells (hPBMCs) with GMMA from *Shigella sonnei*. We studied GMMA both with wild-type hexa-acylated lipid A and with the corresponding less reactogenic penta-acylated form. Using multicolor flow cytometry, we assessed the activation of immune cell subsets and we profiled intracellular cytokine production after GMMA stimulation. Moreover, we measured the secretion of thirty cytokines/chemokines in the cell culture supernatants. Our data indicated activation of monocytes, dendritic, NK, B, and γδ T cells. Comparison of the cytokine responses showed that, although the two GMMA have qualitatively similar profiles, GMMA with modified penta-acylated lipid A induced a lower production of pro-inflammatory cytokines/chemokines compared to GMMA with wild-type lipid A. Intracellular cytokine staining indicated monocytes and dendritic cells as the main source of the cytokines produced. Overall, these data provide new insights into the activation of key immune cells potentially targeted by GMMA-based vaccines.

## Introduction

*Shigella* is a Gram-negative pathogenic bacteria and based on the lipopolysaccharide (LPS) O-antigen structure it is possible to distinguish different bacterial serotypes (Shad and Shad, 2021). Some of these serotypes contribute to the global burden of shigellosis (Liu et al., 2008; Rossi et al., 2014). This infection represents a major global health problem, especially in children of developing countries (Kotloff et al., 2013; Kotloff, 2017), where it causes approximately 200,000 deaths per year (Lozano et al., 2012; Khalil et al., 2018; Shad and Shad, 2021). In addition to this, an increase of multi-drug resistance in different *Shigella* serotypes has been observed in the last decade (Klontz and Singh, 2015; Puzari et al., 2018). In this context, the availability of an effective vaccine remains an urgent need to accelerate disease reduction (Mani et al., 2016).

Gram-negative bacteria are able to naturally release outer membrane vesicles (OMVs), which are mainly composed by lipids, soluble periplasmic components and outer membrane proteins (Bereswill et al., 2012; Mancini et al., 2020). Therefore, OMVs have been proposed as a useful tool for vaccine development. However, low production yields and the fact that they naturally contain LPS with endotoxic activity are major limitations of their use. By altering the LPS structure using genetic modifications, the reactogenicity of OMVs can be significantly reduced (Schromm et al., 2000; Ellis and Kuehn, 2010; Pfalzgraff et al., 2019). Generalized Modules for Membrane Antigens (GMMA) are derived from bacteria containing genetic modifications to induce high level shedding of blebs. In particular, in the case of *Shigella*, the required genetic modification is the deletion of *tolR* gene, encoding for a protein involved in the linking of inner and outer membranes (Bereswill et al., 2012; Rossi et al., 2014). GMMA represent an attractive vaccine technology (Rietschel et al., 1991; Schromm et al., 2000; Bereswill et al., 2012; Rossi et al., 2014; Mancini et al., 2020; Micoli and MacLennan, 2020; Antonelli et al., 2021; Fiorino et al., 2021), as they can naturally express the same bacteria antigens or can be carriers of protein antigens or polysaccharides to induce a specific immune response (Bereswill et al., 2012; Kis et al., 2019; Micoli et al., 2020; Mancini et al., 2021). GMMA stimulate innate immune cells, as they are able to provide innate signals *via* ligands of toll-like receptors (TLRs) and *via* pathogen-associated molecular patterns (PAMPs) (Mancini et al., 2020). Indeed, GMMA have been proven to be recognized by TLR4 and TLR2 of innate immune cells inducing a pro-inflammatory immune response (Koeberling et al., 2014; Rossi et al., 2014; Gerke et al., 2015; Rossi et al., 2016).

Phase I and II clinical studies were carried out on *S. sonnei* GMMA with modified lipid A and were found to be well tolerated up to 100 μg after intramuscular (two or three doses), intradermal or intranasal administrations (Launay et al., 2017; Obiero et al., 2017; Launay et al., 2019). In addition, specific anti-LPS antibodies, which were able to induce complement mediated bactericidal killing in a dose-dependent manner, were found in healthy adults in non-endemic and endemic populations after vaccination (Micoli et al., 2021). Considering their composition and depending on the dose used in a vaccine, GMMA can activate a pro-inflammatory immune response of different intensity. GMMA endotoxicity is mainly associated with the lipid A domain of LPS and its hexa-acylated form is one of the most potent formulations (Munford and Varley, 2006; Rossi et al., 2014; Molinaro et al., 2015; Zariri et al., 2016). In the development of a GMMA-based vaccine for *S. sonnei*, it was demonstrated that the deletion of two genes encoding for late acetyltransferases, either *htrB* (Clementz et al., 1996) or *msbB* (Clementz et al., 1997), resulted in a penta-acylated LPS with reduced endotoxicity (Rossi et al., 2014; Gerke et al., 2015). GMMA from *S. sonnei* with and without these LPS modifications were further investigated, indicating that lipid A mutations reduced the activation of the TLR4 pathway and thus also the levels of key inflammatory cytokines such as IFN-γ, IL-10, IL-12p70, IL-1β, IL-6, TNF-α, and IL-8 (Rossi et al., 2014; Mancini et al., 2020).

In this work, we extended the *in vitro* characterization of the immune response to GMMA derived from a strain of *S. sonnei*, focusing on the innate immune response. Especially, we investigated how *S. sonnei* GMMA with either hexa-acylated (GMMA Hexa) or penta-acylated LPS-lipid A from *htrB* mutant (GMMA Penta) (Gerke et al., 2015) affect cytokines production and cellular activation of human peripheral blood mononuclear cells (hPBMCs).

## Material and Methods

### Peripheral Blood Mononuclear Cell (PBMCs) Stimulation

Human PBMC (hPBMCs) were derived from buffy coats from six healthy donors (Empoli Hospital). Informed consent was obtained before all blood donations. The study protocol conforms to the ethical guidelines of the 1975 Declaration of Helsinki. Before stimulation, cryopreserved hPBMCs were thawed at 37°C and washed twice with a pre-warmed solution (PBS w/o Ca^++^ Mg^++^, Gibco Life Sciences, 2.5 mM EDTA, Euroclone, and 20 µg/ml DNAse, Boheringer Mannheim). hPBMCs were then resuspended in complete medium RPMI-1640 supplemented with 1% non-essential amino acids and 1% sodium pyruvate (Gibco Life Sciences), 1% Penicillin/Streptomycin/Glutamine (Euroclone), and 10% heat-inactivated FBS (Hyclone), and counted to assess cell viability based on Trypan blue dye exclusion (Vi-Cell-XR, Beckman Coulter). The viability of thawed hPBMCs was in the range of 90-98%. After thawing, hPBMCs were seeded at 1x10^6^ live cells/well concentration in round-bottom 96 wells plates (Corning). Cells were then stimulated with positive controls and antigenic stimuli, as follows: *S. sonnei* GMMA Hexa and *S. sonnei* GMMA Penta (GSK Vaccines Institute for Global Health, GVGH) (Gerke et al., 2015) were used at 1 µg/ml for flow cytometry experiments and at 0.01 µg/ml for detection of cytokines in cell culture supernatants; 4 µg/ml CpG (InvivoGen, ODN 2395, Cat. #tlr-2395-1) was used as positive control for B cell subset and plasmacytoid dendritic cells (pDC); 1 µg/ml hexa-acylated LPS form *E. coli* (InvivoGen, LPS-B5 Ultrapure-5mg, Cat. #tlr-pb5lps) was used as positive control for monocytes and myeloid dendritic cells (mDC); and 1μg/ml SEB (Sigma, Cat. S4881-5mg, Lot. 072M4041V) as positive control for T cells and NK cells; complete medium was used as negative control (MED). Cells were incubated for 4 hours or 22 hours at 37°C, 5% CO2.

### Cytokines Determination by the Multi-Plex Technology

Cytokine/chemokine concentration in culture supernatants, collected after 4 or 22 hours of incubation with stimuli, were detected by V-PLEX Human 30-Plex Kit (Meso Scale Discovery) according to manufacturer’s instructions. The following analytes were measured: IFN-γ, IL-10, IL-12p70, IL-13, IL-1β, IL-2, IL-4, IL-6, IL-8, TNF-α, Eotaxin, Eotaxin-3, IL-8HA, IP-10, MCP-1, MCP-4, MDC, MIP-1α, MIP-1β, TARC, GM-CSF, IL-12p40, IL-15, IL-16, IL-17A, IL-1α, IL-5, IL-7, TNF-β, VEGF. All values that were in the detection range were accepted and considered as such. Values above the detection range were replaced with the upper limit of detection. Values below the detection range were replaced with the lower limit of detection. Reported IL-6 values were measured by Human IL-6 Tissue Culture Kit (Meso Scale Discovery) according to manufacturer’s instructions.

### Flow Cytometry: Immunophenotyping and Expression of Activation Markers

For phenotypic characterization of cell subsets and the quantification of activation markers, following 4 or 22 hours of stimulation, cells were stained with Live/Dead Fixable Near-IR Dead Cell Stain Kit (Invitrogen, Cat. L10119) for 20 min at room temperature (RT) in the dark, washed twice with PBS and blocked with 2% rabbit serum in PBS at RT for 20 min. Next, cells were stained with the following monoclonal antibodies (mAbs): anti-CD3 (BUV805), anti-CD11c (BV510), anti-HLA-DR (BUV395), anti-CD4 (BV605), anti-CD8 (PerCP-Cy 5.5), anti-CD14 (BV786), anti-CD16 (BV421), anti-CD19 (PE-Cy5), anti-CD56 (BV650), anti-CD69 (BUV737), anti-CD86 (APC-R700), anti-CD123 (PE-CF594), anti-CD32 (FITC) (BD Biosciences), anti-CD1c (APC), anti-CD40 (BV711), anti-TCRγδ (PE-Cy7), anti-CD64 (PE) (BioLegend) (**Supplementary Table S1**); mAbs were diluted to working dilution recommended by the manufacturer. Cells were incubated at RT for 20 min, washed twice with PBS, and fixed for 20 min at +4°C with Cytofix (BD Bioscience). Next, cells were washed twice with PBS and samples were then acquired on BD LSRFortessa™ X-20 Cell Analyzer configured with up to 5 lasers (blue, violet, red, UV, yellow-green) to detect up to 20 parameters. The instrument was optimized following the procedure described by Perfetto et al. (2006). Data were analysed using FlowJo 10 software (Becton, Dickinson and Company).

### Intracellular Cytokines Detection in hPBMCs Cell Populations by Flow Cytometry

After 2 hours of incubation with stimuli, Brefeldin A (5 μg/ml, BD GolgiPlug) was added to every well and cells were further incubated at 37°C, 5% CO2 for 2 or 20 hours. Then, cells were stained with Live/Dead Fixable Near-IR Dead Cell Stain Kit (Invitrogen, Cat. L10119) for 20 min at RT in the dark, washed twice with PBS and blocked with 2% rabbit serum in PBS at RT for 20 min. Next, cells were surface stained for anti-CD11c (BV510), anti-HLA-DR (BUV737), anti-CD14 (BV786), anti-CD16 (BV421), anti-CD19 (PE-Cy5), anti-CD56 (BV650), anti-CD123 (PE-CF594) (BD Biosciences), anti-TCRγδ (PE-Cy7) (BioLegend). After 20 min of incubation at RT, cells were washed twice with PBS and permeabilized for 30 min at +4°C with CytoFix/CytoPerm (BD Bioscience). Next, cells were washed twice with PermWash (BD Bioscience) and treated with 2% rabbit serum in PBS at +4°C for 20 min to avoid intracellular non-specific binding of antibodies and minimise background signals. Cells were then stained intracellularly with anti-CD8α (APCR700), anti-CD4 (BV605), anti-CD3 (BUV805), anti-TNFα (BUV395), anti-IFNα (APC), anti-MIP1α (PE), anti-IFNγ (BV711) (BD Biosciences), anti-IL6 (PerCP-Cy5.5) (BioLegend) (**Supplementary Table S1**); mAbs were diluted to the working dilution recommended by the manufacturer. Following 20 min of incubation at +4°C, cells were washed twice with PermWash (BD Bioscience). Samples were then acquired on BD LSRFortessa™ X-20 Cell Analyzer configured with up to 5 lasers (blue, violet, red, UV, yellow-green) to detect up to 20 parameters. The instrument was optimized following the procedure described by Perfetto et al. (2006). Data were analysed using FlowJo 10 software (Becton, Dickinson and Company).

### Analysis and Visualization of High-Dimensional Flow Cytometry Data

Flow cytometry data were analysed using FlowJo software 10 (Becton, Dickinson and Company). First, fluorescence intensities were compensated for spectral overlap and biexponentially transformed. Data were then pre-processed to remove debris, doublets, and dead cells. Cell subpopulations were identified using manual gating (**Supplementary Figure S1**). Boxplots comparing the expression of activation markers and of intracellular cytokines were generated using GraphPad Prism v8.0.224 (GraphPad Software, San Diego, California USA, www.graphpad.com). t-distributed stochastic neighbor embedding (t-SNE) (Van der Maaten and Hinton, 2008) was used to visualize flow cytometry data and qualitatively compare the results obtained for the different experimental conditions. As t-SNE is computational demanding, this analysis was run on the minimum number of cells from each Flow Cytometry Standard (FCS) file (i.e. 53,000 cells for every donor and every experimental condition) with default parameters. FlowSOM clustering (Van Gassen et al., 2015) was performed on the down-sampled cells and the results were visualized in the 2D t-SNE map. The self-organizing map was built on a 10x10 grid using a Manhattan distance function. The resulting 100 clusters were then grouped into 7 meta-clusters using consensus hierarchical clustering.

### Statistical Analysis

Nonparametric tests were performed to compare the results obtained for the stimulated and control samples as well as those obtained for different stimuli. To compare the cytokine production between different experimental conditions, the Mann-Whitney test (Mann and Whitney, 1947) was used. P-values were corrected for familywise error rate using the Benjamini-Hochberg method (Benjamini and Hochberg, 1995). Pairwise similarity between cytokine profiles was quantified using the Spearman’s rank correlation coefficient (Daniel, 1990). For flow cytometry data, comparisons were performed using either the Mann-Whitney or the paired Wilcoxon signed rank test (Daniel, 1990). All statistical tests were performed in a two-sided manner using either the SciPy python library v1.5.2 (Virtanen et al., 2020) or GraphPad Prism v8.0.224.

## Results

### *S. Sonnei* GMMA Hexa Induce Higher Production of Cytokines and Chemokines Compared to GMMA Penta in hPBMCs

**Figure 1:**
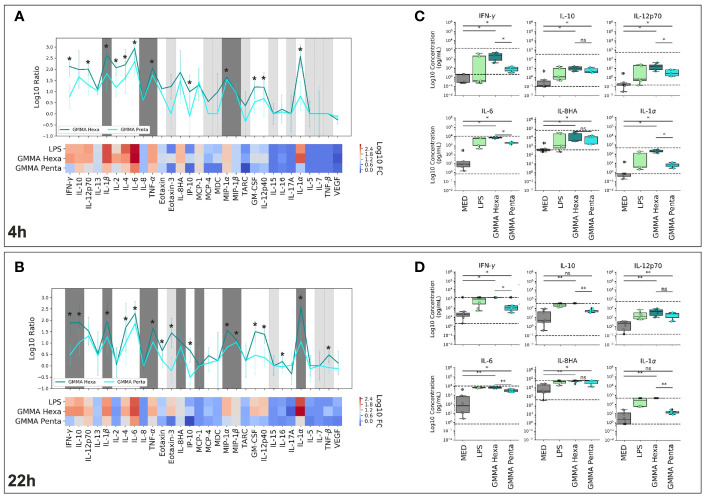
Cytokine levels in the supernatants of hPMBCs stimulated with *S. sonnei* GMMA. **(A, B)** Cytokine expression profile of GMMA Hexa, GMMA Penta, and LPS after 4 hours **(A)** and 22 hours **(B)** incubation. Results are expressed as log_10_ of the median fold change (log_10_FC) over the six donors compared to the non-stimulated samples. Heatmaps show the relative cytokine levels. Line plots further illustrate the comparison between GMMA Hexa (dark cyan) and GMMA Penta (light cyan). Lines mark the median log_10_FC, while error bars represent the standard deviation over the donors. Dark gray and light gray areas indicate cytokines whose expression falls respectively above/below the upper/lower limit of detection (ULOD/LLOD) for more than 50% of the donors. For these cytokines, values were substituted respectively with ULOD or LLOD. **(C, D)** Comparison of cytokine concentrations after 4 **(C)** and 22 hours **(D)** incubation with GMMA Hexa (dark cyan), GMMA Penta (light cyan), or LPS (green). Non-stimulated samples are shown as reference in gray (MED). Dots show the results for each of the donors. Concentrations out of the detection range were replaced with the detection limits. Dashed black lines indicate the upper and lower limits of detection. The Mann-Whitney test with Benjamini-Hochberg correction was used to determine the significance of the observed differences (*p-value < 0,05, **p-value<0.005; ns stands for nonsignificant, p-value > 0.05).

*S. sonnei* GMMA with hexa-acylated wild-type lipid A (GMMA Hexa) and GMMA carrying lipid A modification (GMMA Penta) have been previously shown to trigger in hPBMCs the production of pro-inflammatory cytokines such as IFN-γ, IL-12p70, IL-1β, IL-6, IL-8, and TNFα, but also of the cytokine IL-10 (Rossi et al., 2014). Here, we characterized a broader panel of thirty cytokines and chemokines. The two different GMMA were used to stimulate *in vitro* hPBMCs derived from different donors. Hexa-acylated LPS was used as positive control considering that mutated GMMA differ in the structure of LPS-lipid A. **Figure 1** reports the results obtained after 4 (**Figures 1A, C**) and 22 hours (**Figures 1B, D**) stimulation. In agreement with previous studies (Rossi et al., 2014), we observed a statistically significant increase in the production of IFN-γ, IL-12p70, IL-1β, IL-6, TNF-α, and IL-8HA in response to GMMA, compared to untreated cells after both 4 and 22 hours of treatment (**Figures 1C, D**, and **Supplementary Figures S2**, **3**). In addition, we found that GMMA could induce production of IL-13, IL-4, MIP-1α and MIP-1β at both time points, of IL-10, IL-2, MCP-1, GM-CSF, IL-12p40 and IL-1α only at 4 hours, and of Eotaxin only at 22 hours of stimulation. Spearman’s correlation analysis showed a positive correlation between the cytokine profiles induced by GMMA Hexa and GMMA Penta (Spearman correlation coefficients of 0.8 and 0.76 at 4 and 22 hours, respectively, **Supplementary Figure S4**). Nonetheless, GMMA Hexa stimulated a significantly higher production of many of the investigated cytokines (i.e., IFN-γ, IL-1β, IL-4, IL-6, TNF-α, IP-10, MIP-1α, GM-CSF, IL-12p40, and IL-1α) with respect to GMMA Penta (significant differences are indicated in **Figures 1A, B**). Finally, we found that the cytokine profile induced by stimulation with hexa-acylated LPS correlated more with that obtained with GMMA Hexa (Spearman correlation coefficient of 0.95 both at 4 and 22 hours, **Supplementary Figure S4**) and less with that obtained with GMMA Penta (Spearman correlation coefficients of 0.87 and 0.78 at 4 and 22 hours, respectively, **Supplementary Figure S4**). This is consistent with the *htrB* deletion in GMMA Penta.

### GMMA Predominantly Activate Plasmacytoid Dendritic Cells (pDC), B Cells, γδ T and Natural Killer (NK) Cells

**Figure 2:**
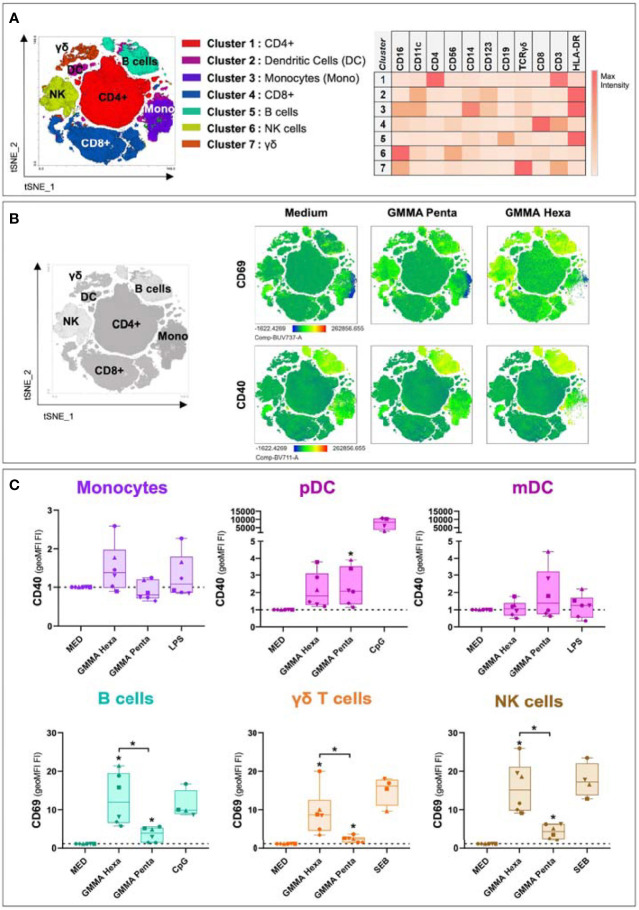
Flow cytometry analysis for activation marker upregulation in different cell populations after 22 hours of stimulation. **(A)** t-SNE projection of the FlowSOM clustering results. Clusters were assigned to cellular subpopulations according to the expression profile of specific population markers (right panel). **(B)** t-SNE plots showing the expression of two different activation markers among different conditions. **(C)** Upregulation levels of CD40 and CD69 in different cellular populations after hPBMCs stimulation with GMMA Hexa, GMMA Penta and specific positive controls. LPS was used as positive control for monocytes and mDC; CpG for B cells and pDC; and SEB for γδ T cells and NK cells. Marker upregulation is expressed as fold increase (FI) of the geoMFI with respect to negative control (MED). Dotted lines indicate the reference value of 1 for the control samples. The different dots represent the six different donors. Significance was estimated using the paired Wilcoxon test (*p < 0.05). Significant differences with respect to the negative control are indicated on top of each box, instead significant differences among stimuli are shown explicitly. Non-significant differences are not shown.

The analysis of cytokines released in cell culture supernatants of hPBMCs showed that *S. sonnei* GMMA Hexa and GMMA Penta induced a pro-inflammatory immune response. Next, to understand which cell populations were activated by these GMMA, we analysed the expression of specific activation markers by flow cytometry. Cell populations were identified both by manual gating and unsupervised clustering. FLOWSOM clustering returned 7 cell clusters which were mapped to the manually gated populations by comparing the expression of 11 surface markers (**Figure 2A**). To gain an overview on the activation profile of the different cell populations, we performed t-SNE dimensionality reduction of the 11-dimensional concatenated flow cytometry data. t-SNE plots showed key regions enriched for expression of activation markers after 22 hours stimulation with GMMA (**Figure 2B**). Upregulation of the cell activation marker CD69 was observed in regions corresponding to B cells, CD4^+^, CD8^+^ and γδ T cells, and NK cells, while upregulation of the costimulatory molecule CD40 was detected in B cells (**Figures 2B, C** and **Supplementary Figure S5**). A quantitative analysis carried out on the manually gated populations confirmed these results and indicated the additional upregulation of CD40 and CD86 in pDC upon stimulation with GMMA Penta and GMMA Hexa, respectively (**Figure 2C** and **Supplementary Figure S5**). Interestingly, the upregulation of these activation markers is significantly higher for GMMA Hexa than for GMMA Penta confirming our previous results analyzing secreted cytokine profiles, except for pDC activation markers. Nonetheless, the residual activity suggests that cellular activation does not depend exclusively on the hexa-acylated LPS. A similar analysis was carried out for the flow cytometry data collected after 4 hours stimulation with GMMA. However, at this time point no significant cellular activation was observed (**Supplementary Figure S6**).

### Multiple Cell Populations SecretePro-Inflammatory Cytokines Upon Stimulation With GMMA

We developed an intracellular cytokine staining panel with the objective to determine which of the identified immune cell populations produced cytokines in response to GMMA. Samples were stained using 16 mAbs indicated in the Material and Methods section to identify specific cell populations producing cytokines in response to GMMA treatment. Specifically, monocytes were identified as CD3^-^CD19^-^CD56^-^HLADR^+^CD14^+^CD16^+/-^ (the three monocytes subsets, classical CD14^++^CD16^-^, intermediate CD14^++^CD16^+^, and non-classical CD14^+^CD16^++^ are included in this gate), T cells as CD3^+^CD4^+^ (CD4^+^ T cells), CD3^+^CD8^+^ (CD8^+^ T cells), and CD3^+^CD4^-^CD8^-^TCRγδ^+^ (γδ T cells), B cells as CD3^-^CD19^+^, NK cells as CD3^-^CD19^-^CD56^bright^CD16^-^ (NKbright) and CD3^-^CD19^-^CD56^dim^CD16^+^ (NKdim), and dendritic cells as CD3^-^CD19^-^CD56^-^CD14^-^HLADR^+^CD11c^+^CD123^-^ (mDC) and CD3^-^CD19^-^CD56^-^CD14^-^HLADR^+^CD11c^-^CD123^+^ (pDC). A graphical depiction of the gating strategy is reported in **Supplementary Figure S1**. Within each subset we measured the frequency of cells producing the cytokines IFN-α, IFN-γ, IL-6, MIP-1α, and TNF-α by using specific-cytokine antibodies conjugated with different fluorochromes.

**Figure 3:**
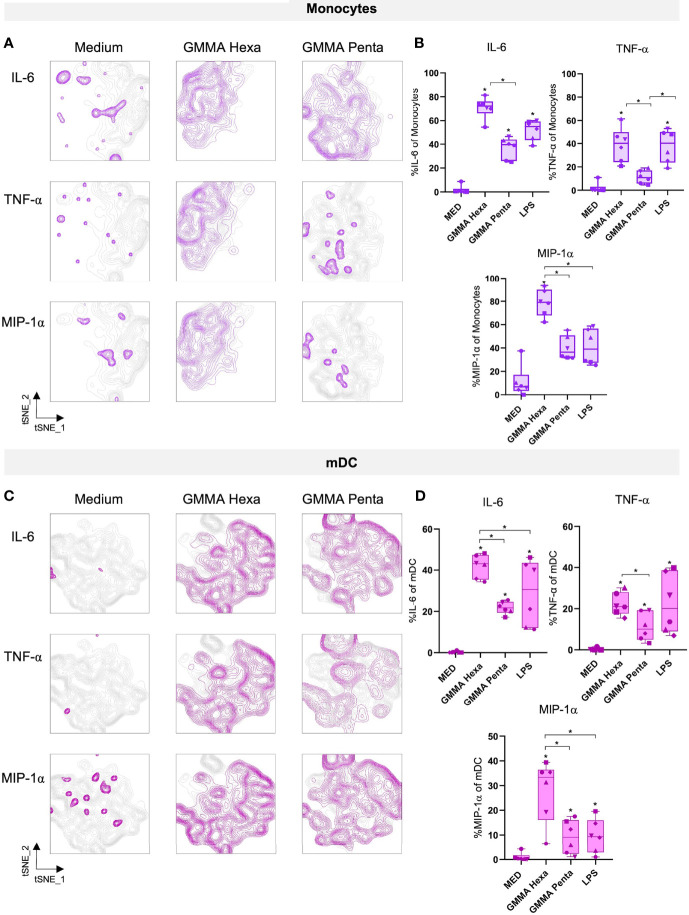
Intracellular cytokines production after 22 hours of stimulation with GMMA. **(A–C)** t-SNE projections of two different cell populations, i.e. monocytes and mDC, showing intracellular cytokines production after stimulation with GMMA Hexa and GMMA Penta. Medium and MED are used as synonyms for control samples. **(B–D)** Percentages of cells producing the analyzed cytokines. Percentage were calculated on the manually gated cell populations. The different dots represent the six different donors. Significance was estimated using the paired Wilcoxon test (*p < 0.05). Significant differences with respect to the negative control are indicated on top of each box, instead significant differences among stimuli are shown explicitly. Non-significant differences are not shown.

Results from both automated t-SNE (**Figures 3A, C**) and manual gating (**Figures 3B, D**) analyses revealed that antigen-presenting cells (APCs), such as monocytes and mDC, produced IL-6, TNF-α and MIP-1α after 22 hours stimulation with either GMMA (**Figure 3** and **Supplementary Figure S7**). Notably, in line with the results of cytokines in culture supernatants, GMMA Penta induced a similar but lower inflammatory profile compared to GMMA Hexa. Moreover, intracellular cytokines production was observed as early as 4 hours after stimulation (**Supplementary Figure S8**).

## Discussion

Outer membrane vesicles (OMVs) released by Gram-negative bacteria resemble the composition of the bacterial outer membrane. Therefore, OMVs are mainly composed by lipids, soluble periplasmic components, outer membrane proteins and antigenic polysaccharides (Mani et al., 2016; Mancini et al., 2020). In order to enhance the blebbing and reduce a possible systemic reactogenicity, genetically modified particles, called GMMA have been developed (Bereswill et al., 2012; Mancini et al., 2020; Micoli et al., 2020). Different studies (Bereswill et al., 2012; Gerke et al., 2015) have shown that through genetic modifications it is possible to reduce reactogenicity of GMMA particles, which thus represent a tool for the generation of vaccines against emerging diseases.

In this study, we investigated in depth the immune response induced by GMMA derived from *S. sonnei*, with or without LPS modification (GMMA Penta and GMMA Hexa, respectively), with a focus on cells of the innate immune system. Our data confirmed that *htrB* deletion modifying the lipid A structure results in a reduced pro-inflammatory immune response (Rossi et al., 2014). Both GMMA Hexa and GMMA Penta induced a predominantly pro-inflammatory immune response in hPBMCs. In agreement with Rossi et al. (2014), we observed the production of IFN-γ, IL-10, IL-12p70, IL-1β, IL-6, TNF-α and IL-8HA. In addition, due to our analysis allowing characterization of a broader panel of cytokines, we found increased levels of IL-2, IL-4, Eotaxin, MCP-1, MIP-1α, MIP-1β, GM-CSF, IL-12p40, and IL-1α at either or both timepoints. Interestingly, we found a positive correlation between the cytokine profiles induced by GMMA Penta and GMMA Hexa from *S. sonnei*. However, GMMA Hexa induced higher production of pro-inflammatory cytokines/chemokines compared to GMMA Penta. In fact, production of IFN-γ, IL-1β, IL-4, IL-6, TNF-α, IP-10, MIP-1α, GM-CSF, IL-12p40, and IL-1α was significantly higher after stimulation with GMMA Hexa. Here, we documented that several immune cell populations were activated by GMMA. Not only classical APCs such as monocytes and dendritic cells, but also B lymphocytes and γδ T lymphocytes upregulated activation markers and induced high levels of cytokines in response to GMMA. Again, GMMA Penta induced a lower level of activation and cytokines production compared to GMMA Hexa. We found that IL-6 and TNF-α were produced mainly by monocytes and mDC. Moreover, we demonstrated that the lipid A genetic modifications do not completely abrogate the immune stimulation of GMMA. It is also probable that residual cytokine production and cellular activation could be due to a non-lipid A related TLR2 activation (Rossi et al., 2014). A possible explanation for the activation by GMMA of less expected immune subsets such as B lymphocytes and γδ T lymphocytes, could be related to the expression of TLR4 and TLR2 on their surface, that are stimulated by LPS and lipoproteins, respectively. It has indeed been previously demonstrated that CD138^+^ B cells (Browne, 2012; Mancini et al., 2020) and γδ T cells (Vijay, 2018) can express TLRs, particularly when activated. Although lipid A detoxification is associated with a reduced immune response of innate immune cells, GMMA Penta still maintain an activation pattern that correlates with that induced by GMMA Hexa. This can ensure a proper activation of the innate system which can result in maintaining profile of adjuvanticity while also guaranteeing an acceptable safety profile (Launay et al., 2017; Obiero et al., 2017; Launay et al., 2019). A molecular and cellular synergy may be beneficial to the outcome of vaccination, improving a protective response to the pathogens. Furthermore, it has been previously reported that lymphocytes activation by TLRs ligands, like LPS, can induce strong B cells proliferation and IL-6 production (Venkataraman et al., 1999). In line with these results, we showed that *in vitro* stimulation with GMMA can induce upregulation of CD40 and CD69 on B cells, likely due to the presence of TLR4 and/or TLR2 ligands in GMMA. How B cells could complement the information from TLRs with antigen-specific activation through B cell receptors (BCRs), and T-cell help through CD40, needs to be further investigated. Finally, γδ T lymphocytes have also been shown to express TLRs on their surface when the cells are activated, playing an active role in the detection of pathogens and associated PAMPs, as well as damage-associated molecular pattern (DAMPs) directly (Vijay, 2018). Blocking of TLRs on cell subsets by antagonist molecules in *in vitro* experiments will help to clarify the contribution of specific TLRs in the residual ability to induce innate responses to GMMA carrying modified lipid A, as previously shown for both *Shigella* (Rossi et al., 2014) and *Salmonella* (Rossi et al., 2016). We also observed that the activation of pDC was not abrogated by lipid A modification, suggesting that their activation is TLR4 independent. A possible speculation could be that the presence of residual DNA from GMMA preparation activates the TLR9 pathway. In-depth experiments will be needed for unravelling activation mechanisms of this cell population.

With the caveat that hPBMCs from only six healthy donors were used for this *in vitro* analysis, we found that monocytes, dendritic, NK, B, and γδ T cells were significantly activated by GMMA. These results suggest that the ability of GMMA to activate cells is independent of donors’ individual characteristics. However, further validation in a larger number of donors is needed to confirm these findings and provide statistical evidence for other target cell populations which seem to be activated by GMMA but lack statistical significance. Moreover, investigations on sorted cell populations will evaluate whether cellular activation is triggered directly by GMMA *via* cellular receptors or mediated indirectly *via* the microenvironment induced by GMMA. Overall, the data presented here provide new insights into the activation of key human immune cells targeted by GMMA based vaccines that could enhance the magnitude and the persistence of the antigen specific immune response and the resulting protection from infection.

## Data Availability Statement

The original contributions presented in the study are included in the article/**Supplementary Material**. Further inquiries can be directed to the corresponding author.

## Ethics Statement

The studies involving human participants were reviewed and approved by Empoli Hospital, IT. The patients/participants provided their written informed consent to participate in this study.

## Author Contributions

FS, CB, LM, OR, FM, EB, MB, and CU were involved in the conception and design of the study. STo performed the study. STo, CS, and STa were involved in acquisition and generation of flow cytometry data. STo, BC, CE, and FS were involved in data analysis and data interpretation. All authors were involved in drafting the manuscript or revising it critically for important intellectual content. All authors had full access to the data and approved the manuscript before it was submitted by the corresponding author.

## Funding

This work was entirely sponsored by GlaxoSmithKline Biologicals SA. GSK was also responsible for all costs incurred in publishing.

## Conflict of Interest

STo is a student at the University of Siena and participated in a post graduate studentship program at GSK. BC, CE, CS, STa, MB, CB, and FS are employees of GSK group of companies. OR, FM, and LM are employees of the GSK Vaccines Institute for Global Health Srl, an affiliate of GlaxoSmithKline Biologicals SA.

The remaining authors declare that the research was conducted in the absence of any commercial or financial relationships that could be construed as a potential conflict of interest.

This work was sponsored by GlaxoSmithKline Biologicals SA which was involved in all stages of the study conduct and analysis.

## Publisher’s Note

All claims expressed in this article are solely those of the authors and do not necessarily represent those of their affiliated organizations, or those of the publisher, the editors and the reviewers. Any product that may be evaluated in this article, or claim that may be made by its manufacturer, is not guaranteed or endorsed by the publisher.
