# Supplementary material for: Dissecting in Vitro the Activation of Human Immune Response Induced by Shigella sonnei GMMA
Source: Front Cell Infect Microbiol. 2022 Feb 3;12:767153. doi: 10.3389/fcimb.2022.767153 (PMC8851470; doi:10.3389/fcimb.2022.767153)
Supplement: Supplementary file 1 [file DataSheet_1.docx]

Supplementary Material

## Supplementary Figures and Tables

## Supplementary Tables

**Supplementary Table S1. Table of antibodies used in flow cytometry and respective fluorochrome and clone.**

| **Antibody** | **Fluorochrome** | **Clone** |
| --- | --- | --- |
| LIVE/DEAD™ Fixable Near-IR (Invitrogen) | Near-IR fluorescent reactive dye | / |
| Anti-CD3 (BD Bioscience) | BUV 805 | UCHT1 |
| Anti-CD4 (BD Bioscience) | BV605 | RPA-T4 |
| Anti-CD8 (BD Bioscience) | PerCP-Cy5.5 | SK1 |
| Anti-TCRγδ (BioLegend) | PE-Cy7 | B1 |
| Anti-CD19 (BD Bioscience) | PE-Cy5 | HIB19 |
| Anti-CD11c (BD Bioscience) | BV510 | B-ly6 |
| Anti-HLA DR (BD Bioscience) | BUV395 | G46-6 |
| Anti-CD14 (BD Bioscience) | BV786 | M5E2 |
| Anti-CD16 (BD Bioscience) | BV421 | 3G8 |
| Anti-CD32 (BD Bioscience) | FITC | FLI8.26 |
| Anti-CD64 (BioLegend) | PE | 10.1 |
| Anti-CD56 (BD Bioscience) | BV650 | NCAM16.2 |
| Anti-CD1c (BioLegend) | APC | L161 |
| Anti-CD123 (BD Bioscience) | PE-CF594 | 7G3 |
| Anti-CD86 (BD Bioscience) | APC-R700 | 2331 (FUN-1) |
| Anti-CD40 (BioLegend) | BV711 | 5C3 |
| Anti-CD69 (BD Bioscience) | BUV737 | FN50 |
| Anti-HLA-DR (BD Bioscience) | BUV737 | G46-6 |
| Anti-CD8α (BD Bioscience) | APC-R700 | SK1 |
| Anti-TNFα (BD Bioscience) | BUV395 | Mab11 |
| Anti-IFNα (BD Bioscience) | Alexa Fluor 647 | 7N4-1 |
| Anti-MIP1α (BD Bioscience) | PE | CR3M |
| Anti-IFNγ (BD Bioscience) | BV711 | B27 |
| Anti-IL6 (BioLegend) | PerCP-Cy5.5 | MQ2-13A5 |

## Supplementary Figures

**Supplementary Figure S1. Gating strategy for flow cytometry experiments.** *From left to right and from up to down: (A) LIVE/DEAD Fixable Near-IR Dead Cell Stain Kit was used to identify live cells, FSC x SSC gating to select cells based on size and granularity, and SSC-W x SSC-A to select single cells. (B-F) Staining with specific antibodies to gate cell populations: (B) CD3, CD56 and CD16 for NK cells; (C) CD3 and CD19 for B cells; (D) CD3, CD19, CD56, CD14, HLA-DR, CD11c and CD123 for dendritic cells; (E) CD3, CD19, CD56, CD14, HLA-DR, CD14 and CD16 for monocytes; and (F) CD3, CD4, CD8 and TCR-γδ for T cells.*


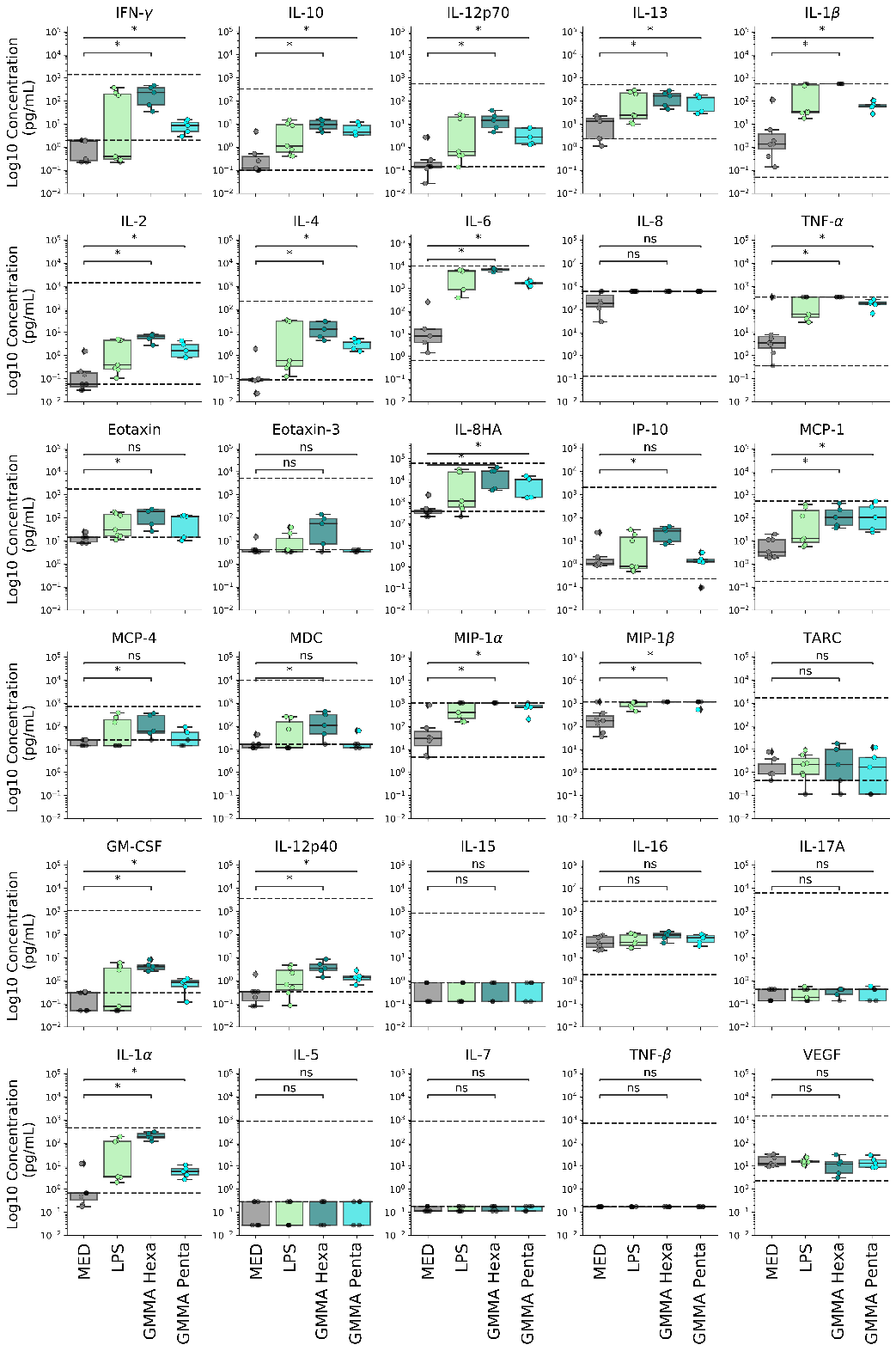


**Supplementary Figure S2.** Cytokine levels in the supernatant of hPBMCs stimulated with GMMA S. sonnei for 4 hours. *Cytokine concentrations were measured after 4 hours incubation with GMMA Hexa (dark cyan), GMMA Penta (light cyan), or LPS (green). Non-stimulated samples are shown as reference in gray (MED). The Mann-Whitney test with Benjamini-Hochberg correction was used to determine the significance of the observed differences (*p-value < 0,05, **p-value<0.005; ns stands for nonsignificant, p-value > 0.05). Dashed black lines at the top of every panel indicate the upper limit of detection while dashed black lines at the bottom indicate the lower limit of detection. Dots show the results for each of the six donors. Concentrations out of the detection range were replaced with the detection limits. The results for IFNγ, IL-1α, IL-6, IL-8HA, IL-10 and IL-12p70 are the same as those in Figure 1C in the main text.*


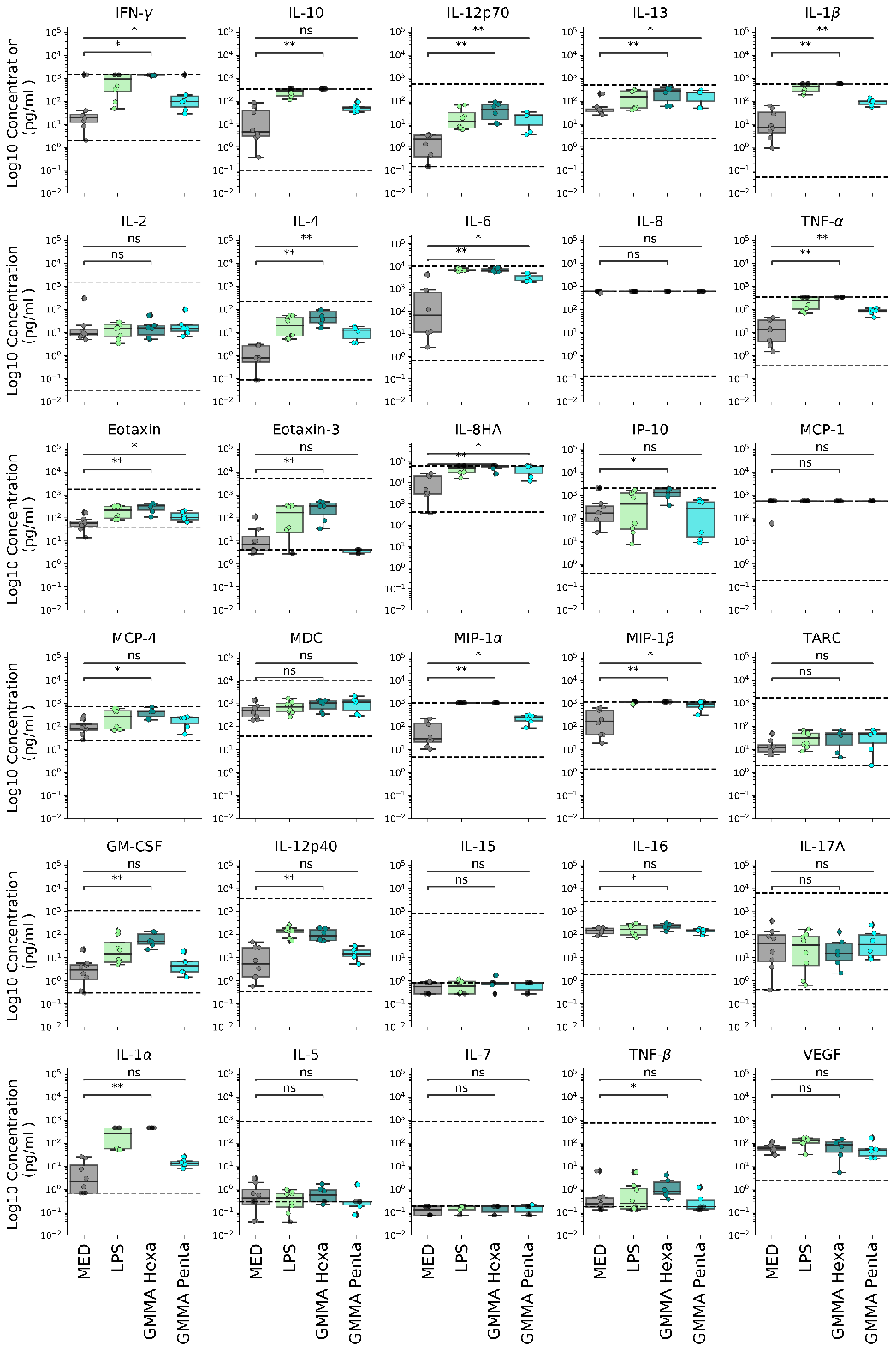


**Supplementary Figure S3.** Cytokine levels in the supernatant of hPMBCs stimulated with GMMA S. sonnei for 22 hours. *Same panel of Figure S3 except that cytokine concentrations were measured after 22 hours incubation with GMMA Hexa (dark cyan), GMMA Penta (light cyan) or LPS (green). The results for IFNγ, IL-1α, IL-6, IL-8HA, IL-10 and IL-12p70 are the same as those reported in Figure 1D in the main text.*


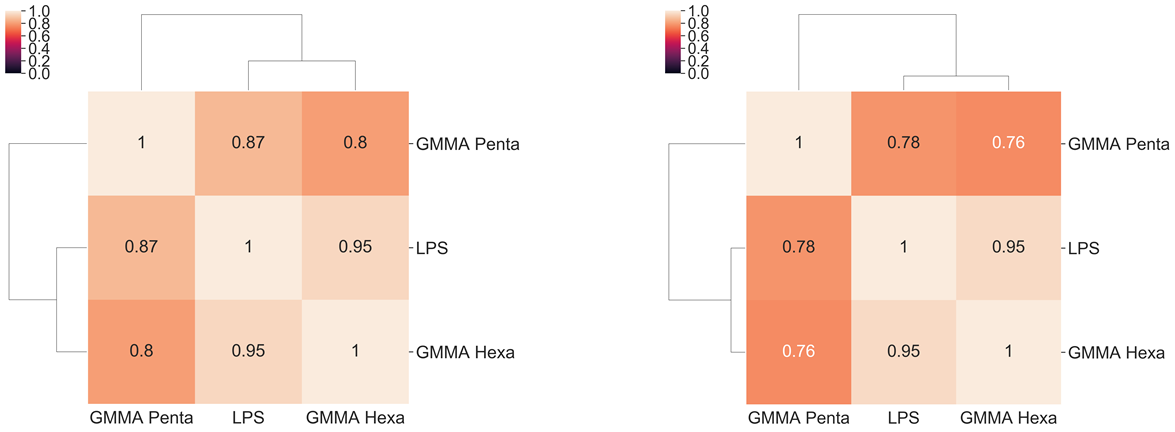


**Supplementary Figure S4.** **Similarity between the cytokine profiles in the supernatants of hPBMCs.** *Heatmap of the Spearman correlation coefficient between the cytokine expression profiles of the different stimuli after 4 hours (A) and 22 hours (B) of incubation. Expression profiles were obtained as the median fold change over the donors compared to the non-stimulated samples (see Figure 1 in the main text). Pairwise comparisons between GMMA Hexa, GMMA Penta, and LPS are shown. Hierarchical clustering was performed on top of the heatmap to sort stimuli by their similarity.*

**Supplementary Figure S5.** **Flow cytometry analysis of surface activation markers for different cell populations after 22 hours incubation with GMMA.** *Comparison of the Log10 MFI of different activation markers among different cellular populations stimulated with GMMA Hexa, GMMA Penta, or specific positive controls. Non-stimulated samples are shown as reference in gray (MED). Positive controls specific for each population were used, i.e. LPS for monocytes and mDC, SEB for T cells and NK cells, and CpG for B cells and pDC. The experiment was conducted once for each of the six different donors and dots represent the results for each of the donors. Significance was estimated using the paired Wilcoxon test. However, no significant difference was observed.*


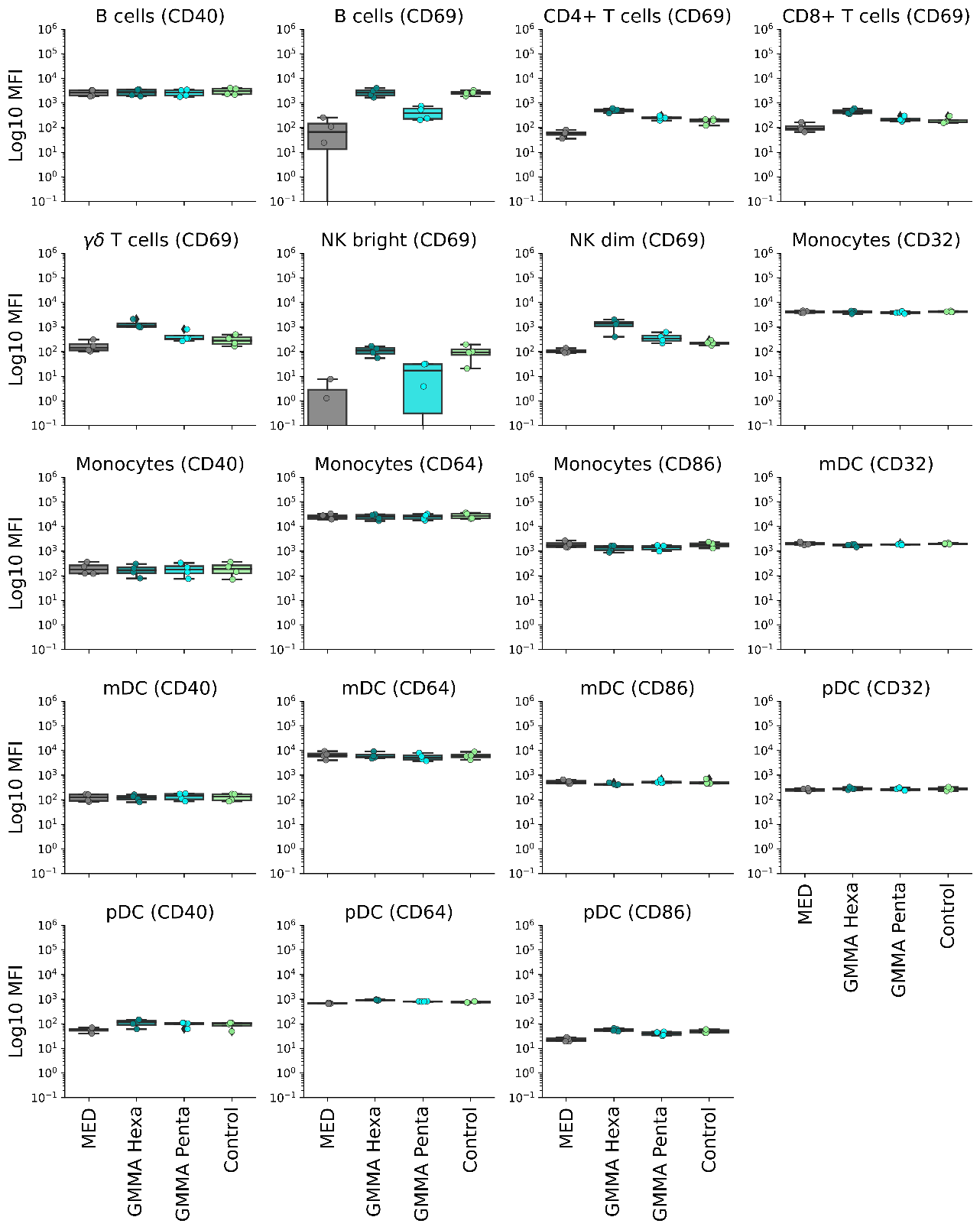


**Supplementary Figure S6.** **Flow cytometry analysis of surface activation markers for different cell populations after 4 hours incubation with GMMA.** *This is the same as Figure S5 except that flow cytometry data were collected after 4 hours hPBMCs stimulation with GMMA Hexa, GMMA Penta, or specific positive controls. Significance was estimated using the paired Wilcoxon test (*p <0,05; ns stands for nonsignificant, p- value > 0.05).*


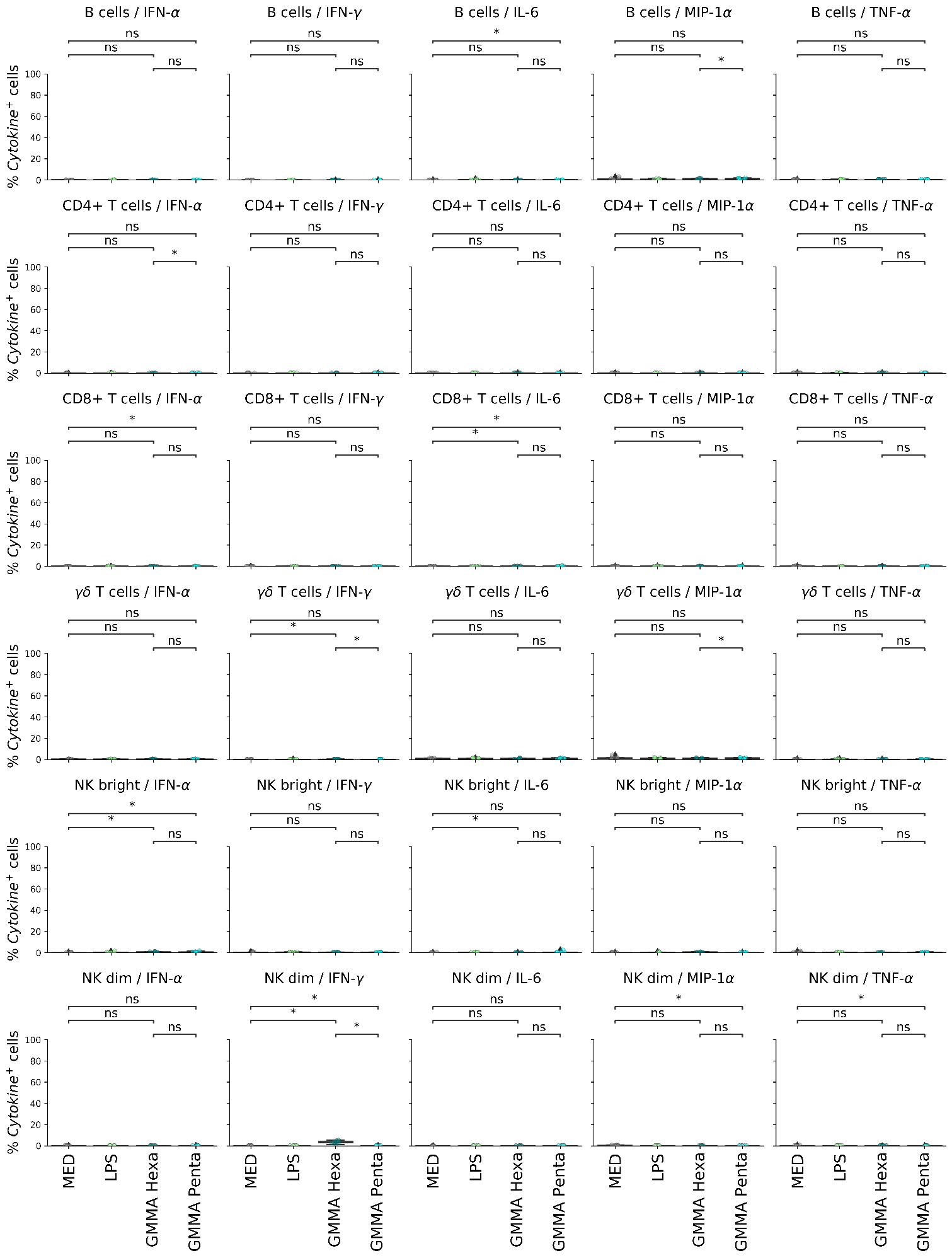


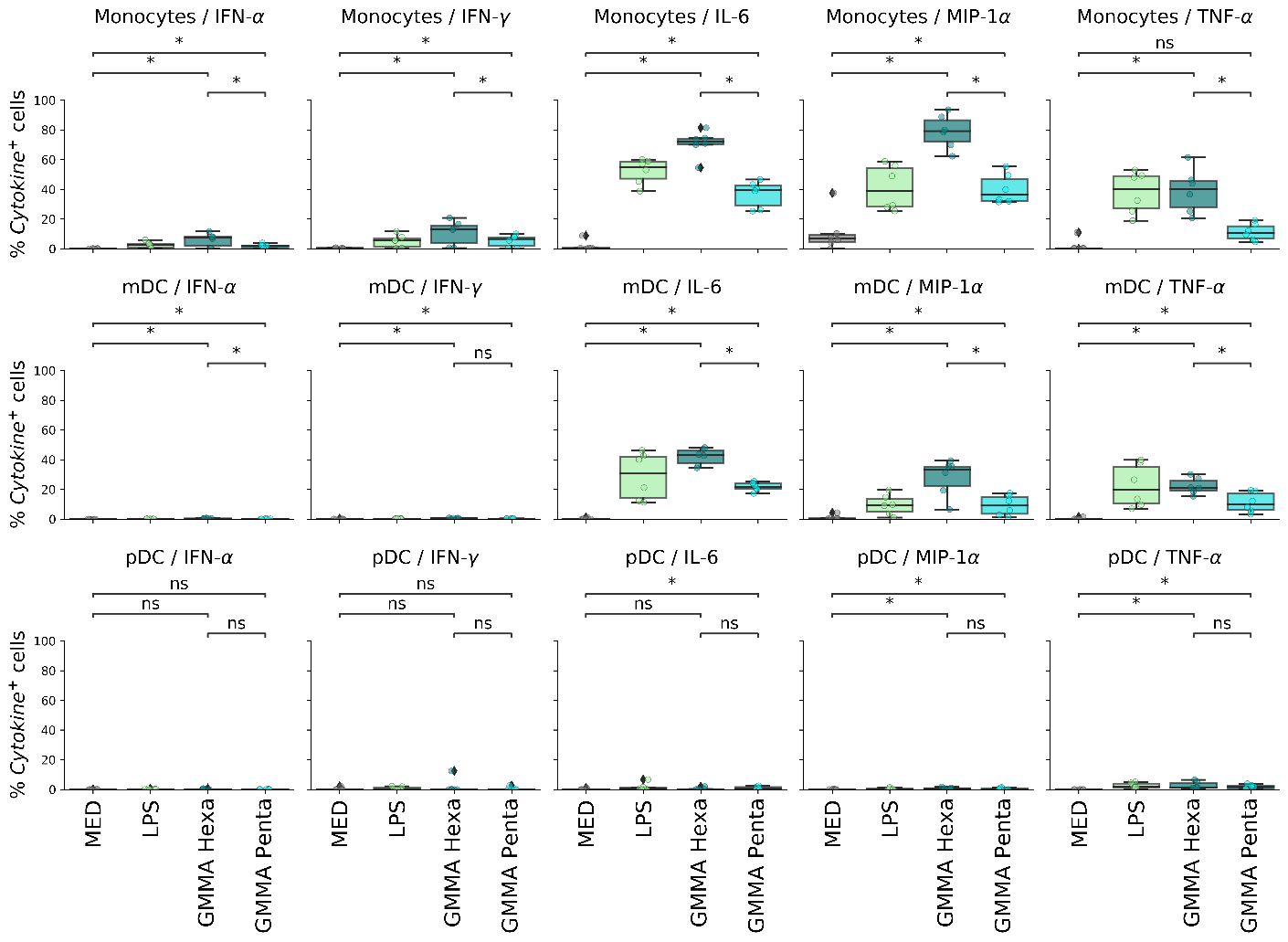


**Supplementary Figure S7.** **Flow cytometry analysis for intracellular cytokines production in different cell populations after 22 hours incubation with GMMA.** *Percentages of cells producing the analyzed cytokines after 22 hours stimulation. Dots represent the results for each of the six donors. Significance was estimated using the paired Wilcoxon test (*p <0,05; ns stands for nonsignificant, p-value > 0.05).*

*
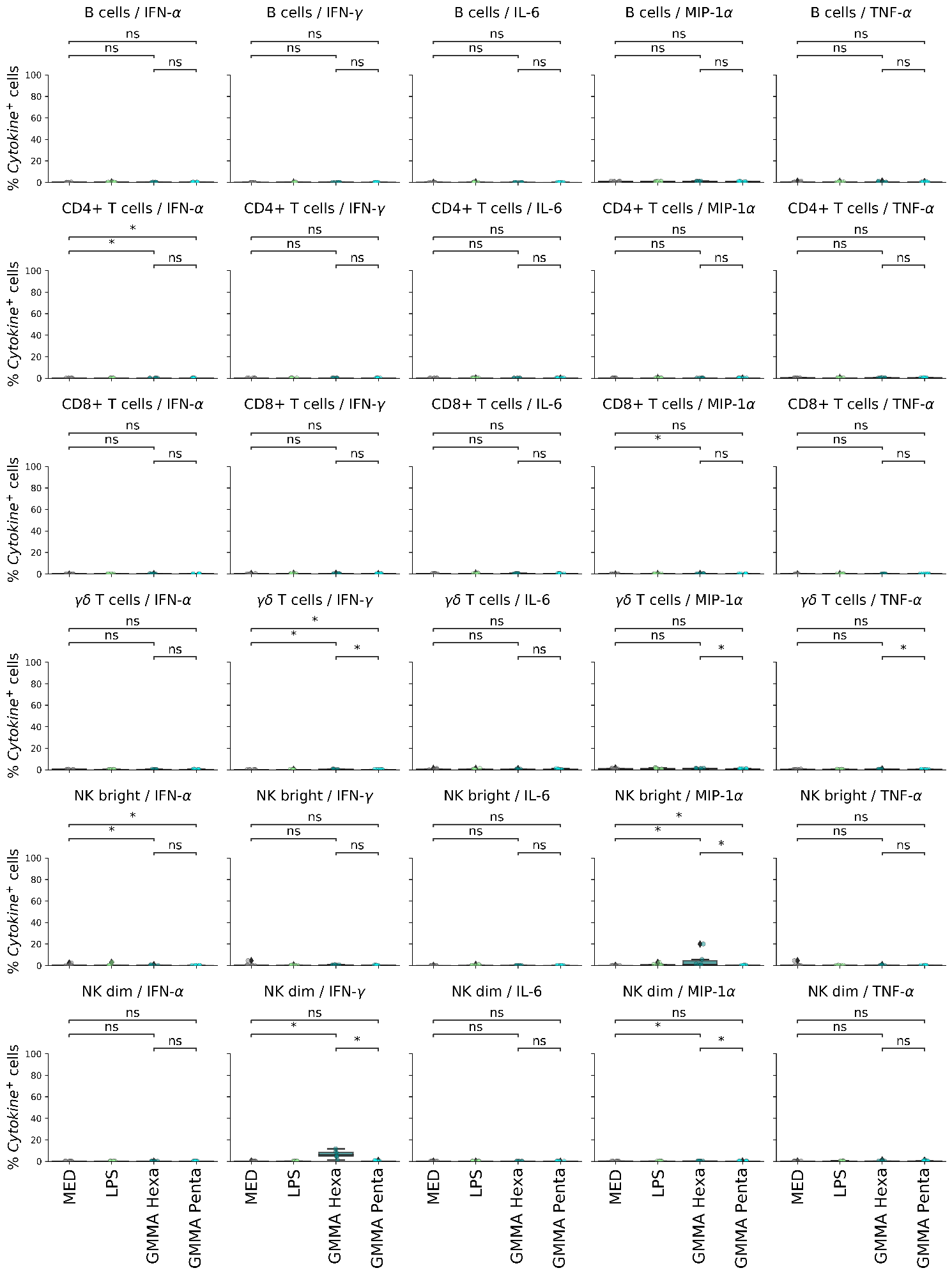
*

*
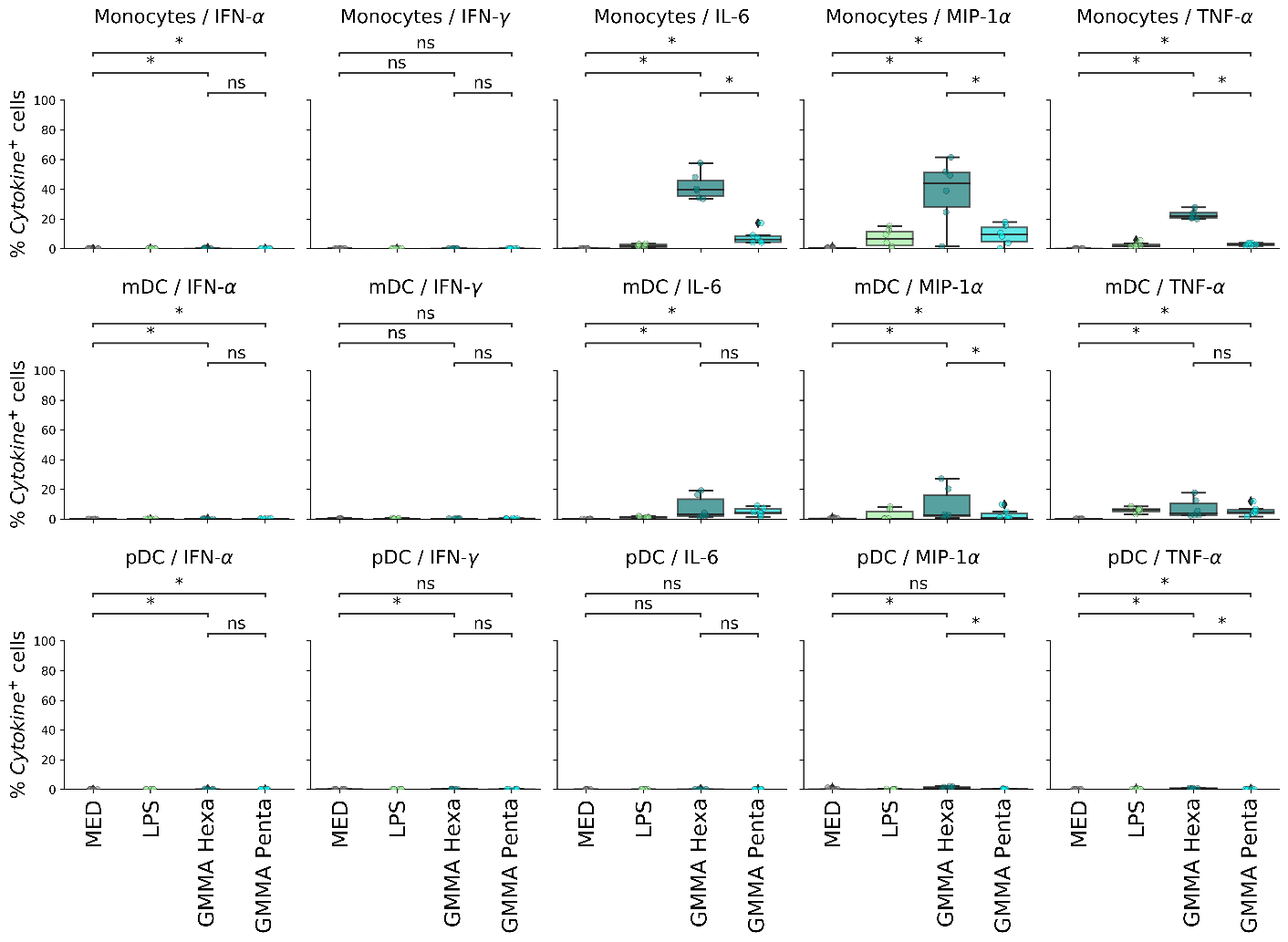
*

**Supplementary Figure S8.** **Flow cytometry analysis for intracellular cytokines production in different cell populations after 4 hours incubation with GMMA.** *This is the same as Figure S7 except that flow cytometry data were collected after 4 hours stimulation.*
